# *Angiostrongylus cantonensis* Galectin-1 interacts with Annexin A2 to impair the viability of macrophages *via* activating JNK pathway

**DOI:** 10.1186/s13071-020-04038-w

**Published:** 2020-04-08

**Authors:** Xiaomeng Shi, Mengran Xiao, Zhiyue Xie, Qing Shi, Yuanjiao Zhang, Jianmei W. Leavenworth, Baolong Yan, Huicong Huang

**Affiliations:** 1 Department of Parasitology, School of Basic Medical Sciences, Wenzhou Medical University, Wenzhou, 325035 Zhejiang People’s Republic of China; 2 The First Affiliated Hospital of Wenzhou Medical university, Wenzhou, 325035 Zhejiang People’s Republic of China; 3 The First Clinical College, Southern Medical University, Guangzhou, 510515 Guangdong People’s Republic of China; 4 Department of Neurosurgery, University of Alabama at Birmingham, Birmingham, AL USA; 5 Department of Microbiology, University of Alabama at Birmingham, Birmingham, AL USA

**Keywords:** *Angiostrongylus cantonensis*, Galectin-1, Macrophage apoptosis, Annexin A2, JNK signaling

## Abstract

**Background:**

*Angiostrongylus cantonensis* can cause severe symptoms of central nervous system infections. In the host, this parasite localizes in the blood and cerebrospinal fluid, and its secreted components can impact immune responses. Our previous study demonstrated that immune responses were inhibited in *A. cantonensis*-infected mice immunized with Ac-Galectin-1 (AcGal-1). However, the mechanisms by which AcGal-1 regulates the immune responses remain unclear. Macrophages are innate immune cells that rapidly respond to infection. The direct impact of AcGal-1 on macrophages may affect the immune responses.

**Methods:**

AcGal-1 protein was purified by nickel ion affinity chromatography. The effect of AcGal-1 on the apoptosis of macrophages was detected using CCK-8 assay, flow cytometry and western blot. Macrophage membrane proteins bound to AcGal-1 were obtained using the His-tag-based pull-down assay and identified *via* mass spectrometry. Co-localization of AcGal-1 and the macrophage membrane protein Annexin A2 was observed by immunofluorescence microscopy, and their interaction was validated by co-immunoprecipitation experiments. SiRNA-mediated knockdown of Annexin A2 was used to determine if AcGal-1-induced macrophage apoptosis required interaction with Annexin A2. The phosphorylation level of apoptotic signal pathway protein was detected by phospho-antibody microarray and western blot.

**Results:**

Our study showed that AcGal-1 caused apoptosis of the macrophages. AcGal-1 increased the expression of apoptosis proteins caspase-3, caspase-9, Bax, but reduced the expression of anti-apoptosis protein Bcl-2. AcGal-1 interacted with the membrane protein Annexin A2, and knockdown of Annexin A2 expression increased Bcl-2 but decreased Bax levels in AcGal-1-treated cells. Moreover, AcGal-1 increased JNK phosphorylation and the inhibition of JNK phosphorylation in AcGal-1-treated cells decreased the expression of caspase-3, -9, Bax and almost restored Bcl-2 to the level observed in control cells.

**Conclusions:**

AcGal-1 can induce the apoptosis of macrophages by binding to Annexin A2 and activating JNK downstream the apoptotic signaling pathway.
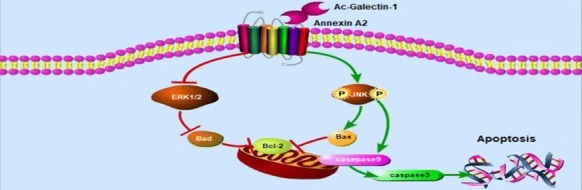

## Background

*Angiostrongylus cantonensis* is a zoonotic pathogen that causes human eosinophilic meningitis [1]. Humans can be infected by accidental ingestion of undercooked intermediate hosts (e.g. *Pomacea canaliculata* and *Achatina fulica*) or third-stage larvae (L3) in drinking water [2]. After penetrating the intestinal wall, *A. cantonensis* transits *via* the circulation to the blood-brain barrier, traverses it and then affects the central nervous system [3]. Larvae in the brain tissue of infected individuals can cause brain and spinal cord symptoms such as headache, fever, vomiting, lethargy, stiff neck, and increased cerebrospinal fluid pressure [4, 5]. Once in the body, *A. cantonensis* can survive in the blood and cerebrospinal fluid for an indefinite period of time by evading the host immune response [6], although the underlying mechanisms remain unclear.

*Angiostrongylus cantonensis* was previously shown to cause apoptosis and necroptosis in the brains of infected mice; this was associated with elevated cleaved caspase-3, -4, and -6, and receptor-interacting serine/threonine-protein kinase (RIP)3 mRNA levels, RIP3, and phosphorylated (p)RIP3 protein levels relative to the levels observed in control mice. Furthermore, apoptotic and necrotic microglia, astrocytes, and neurons were observed in the parenchymal and hippocampal regions of infected mice [2].

In our previous study, using differential proteomics analysis of *A. cantonensis* at different stages of development, we showed that the expression level of *A. cantonensis* galectin (AcGal)-1 was higher in fifth-stage larvae (L5) than in third-stage larvae (L3) [7]. Galectins (Gals) constitute a family of lectins conserved across many species and are characterized by an affinity for β-galactoside and the presence of a conserved sequence motif known as the carbohydrate recognition domain (CRD) [8]. Gals are secreted by cells *via* an unconventional mechanism [9] and play a critical role in apoptosis, cell proliferation, inflammation, immune response, and cell adhesion and migration [10–18]. Parasite Gals have a sequence and structure similar to those of mammalian homologs and are presumed to participate in host-parasite interactions. Gals enable immune evasion by inhibiting the proliferation and activation of immune cells or by causing their death [19]. For example, the binding of Gal to transmembrane protein 147 receptor of peripheral blood mononuclear cells (PBMCs) increases the transcription of Toll-like receptor (TLR)-1, TLR-3 and TLR-4, and the downstream effectors myeloid differentiation primary response 88 (MyD88) and Fas-associated with death domain protein (FADD); the simultaneous activation of both the TLR and caspase pathways induces PBMC apoptosis [19]. The interaction of Gal with PBMCs also promotes the expression of voltage-dependent anion-selective channel protein 2 and induces mitochondrial apoptosis [20]. These findings suggest that AcGal-1 can induce apoptosis.

Our previous study demonstrated that immune responses were inhibited in *A. cantonensis*-infected mice immunized with Gal-1[21]. We speculated that *A. cantonensis* evades the host immune response by secreting AcGal-1 to induce the apoptosis of immune cells including macrophages, which are the first line of defense against infection [22]. To test this hypothesis, we evaluated the proliferation and apoptosis of macrophages derived from a human acute monocytic leukemia line (THP-1) cells treated with AcGal-1. Our results provide a basis for investigating host immune regulation by *A. cantonensis*.

## Methods

### Expression and purification of AcGal-1 fusion protein

Following the protein purification method of Li et al. [23], we used the pCold-III-Gal-1 vector (the empty pCold-III vector or the recombinant construct of AcGal-1) constructed by these authors, which was transformed into *Escherichia coli* BL21 cells. When constructing the plasmid, we added a His-tag to the primer sequence. His-tagged Gal-1 protein expression was induced with 0.1 mM isopropyl-β-d-thiogalactoside and 100 µg/ml ampicillin at 15 °C for 22 h in Luria-Bertani medium. Recombinant protein was purified by Ni-NTA His-Bind-Resin (Merck, Darmstadt, Germany) and verified by sodium dodecyl sulfate-polyacrylamide gel electrophoresis (PAGE). The BCA assay (Beyotime Institute of Biotechnology, Shanghai, China) was used to measure the protein concentration, following standard protocol.

### Cell culture

THP-1 monocytes were cultured in Roswell Park Memorial Institute-1640 medium supplemented with 10% fetal bovine serum and 1 mM sodium pyruvate. Cells were maintained at a density of 5–8 × 10^7^ cells/ml and sub-cultured every 3–4 days to ensure that they remained in the logarithmic growth phase. Cells were seeded into 6-well plates (9.5 cm^2^) at 10^6^–10^7^ cells/well. To induce differentiation, the cells were treated with 50 nM phorbol 12-myristate 13-acetate (PMA) for 48 h, and the medium was replaced with PMA-free culture medium. Cells were cultured in a fully humidified atmosphere at 37 °C and 5% CO_2_. To verify the differentiation of THP-1 cells into macrophages by PMA, the cells were stained with a fluorescent CD11b antibody [24, 25] (Cat# MAB1124-100; R&D Systems, Minneapolis, MN, USA) and analyzed by immunofluorescence microscopy.

### Cell proliferation assay

Cell proliferation was evaluated with a Cell Counting Kit (CCK)-8 (Beyotime Institute of Biotechnology) according to the manufacturer’s instructions. Briefly, cells were seeded in 96-well plates at 37 °C and 5% CO_2_. CCK-8 reagent was added to the wells and the absorbance was measured at 450 nm on a microplate reader.

### Annexin V/propidium iodide (PI) double-staining and flow cytometry

Apoptotic cells were quantified with an Annexin V/fluorescein isothiocyanate (FITC) Apoptosis Detection Kit (Solarbio, Beijing, China) and analyzed by flow cytometry. Macrophages differentiated from THP-1 cells were seeded in 6-well plates at a density of 10^6^–10^7^ cells/well and treated with different concentrations of AcGal-1 (0.5 µg/ml, 1 µg/ml, 1.5 µg/ml, or 2 µg/ml) for 12 h at 37 °C and 5% CO_2_. The cells were washed with phosphate-buffered saline (PBS) and resuspended in 500 µl binding buffer. Annexin V/FITC (5 µl) and PI (5 µl) were added, and the mixture was incubated for 10 min at 37 °C in the dark. After incubation the cells were immediately analyzed by flow cytometry (BD FACSCanto II; BD Biosciences, Franklin Lakes, NJ, USA), with bovine serum albumin (BSA) and dexamethasone used as negative and positive controls, respectively.

### Determination of caspase-3 activity

Adherent cells were enzymatically dissociated with trypsin and resuspended in fresh cell culture medium. The cells were collected by centrifugation at 600×*g* and 4 °C, washed, and lysed on ice for 15 min at a ratio of 100 µl lytic solution to 2 × 10^6^ cells. After adding 2 mM N-acetyl-Asp-Glu-Val-Asp-p-nitroanilide (Ac-DEVD-pNA) (Beyotime Institute of Biotechnology, Shanghai, China), cells were incubated at 37 °C for 1–2 h. The absorbance was measured at 405 nm and subtracted from that of the blank control; this corresponded to the absorbance of pNA produced by caspase-3 catalysis in the sample. The amount of pNA produced in the sample was calculated from a standard curve.

### Plasma membrane protein extraction

Plasma membrane protein concentrations were quantified with a Plasma Membrane Protein Extraction Kit (BioVision, San Francisco, CA, USA). Cells (0.2–10 × 10^8^) were collected in PBS and then centrifuged at 700×*g* for 5 min at 4 °C. The pellet was washed with 3 ml ice-cold PBS and resuspended in 2 ml of homogenization buffer mix (provided in the Plasma Membrane Protein Extraction Kit), and the cells were homogenized 30–50 times on ice with a Dounce homogenizer. The homogenate was centrifuged at 700 ×*g* for 10 min at 4 °C, and the supernatant was transferred to a new tube and centrifuged at 10,000×*g* for 30 min at 4 °C. The pellet contained total cell membrane proteins (including those of the plasma membrane and organelle membranes).

### Pull-down assay

The pull-down assay was performed with the Magpoins^TM^ His-Tag Immunoprecipitation Kit (Sino Biological, Beijing, China). Cells were lysed in Nonidet (N)P40 buffer, the lysate and His-AcGal-1 were mixed in pull-down buffer along with the bead/protein complex (reagents provided in the Magpoins^TM^ His-Tag Immunoprecipitation Kit). The mixture was incubated on a rotary mixer for 10 min at room temperature and the tube was then placed on a magnetic separator for 1 min. The supernatant was discarded, and the beads were washed four times with 300 µl binding/wash buffer before adding 100 µl His-elution buffer. The suspension was incubated on a roller for 5 min at room temperature. The metal beads on the tube wall were collected using a magnetic separator and the supernatant, which contained the eluted His-tag protein and its interaction partners, was transferred to a clean tube. Protein products were analyzed by liquid chromatography-mass spectrometry (LC-MS) and western blotting.

### LC-MS/MS

Each protein fraction (30 μg) was mixed with 200 µl of 8 M urea in 150 mM Tris-HCl (pH 8.5) (UA buffer) in the 30 kDa molecular weight cut-off filter unit (Millipore, MA, USA); any remaining SDS was exchanged by urea in a second washing step with 200 µl of UA buffer. The samples were then diluted with 100 µl IAA (50 mM IAA in UA) and incubated at room temperature for 30 min. These samples were mixed with 100 µl of 25 mM NH_4_HCO_3_ and centrifuged; this step was repeated twice. Each sample was diluted with 40 µl NH_4_HCO_3_ (25 mM) and digested with trypsin at 37 °C for 18 h. After digestion, the peptides were recovered by centrifugation in a new collection tube. The acidified tryptic peptides were sampled on a SC001 traps reverse phase C18 trap column and separated on a SC200 reverse phase C18 nanocolumn (Easy-nLC1000 system; Thermo/Finnigan, San Francisco, CA, USA) *via* a gradient. The mobile phases A and B were 0.1% formic acid in 2% acetonitrile and 0.1% formic acid in 84% acetonitrile, respectively. The LC system was coupled to an orbitrap Q Exactive mass spectrometer (Thermo/Finnigan). The MS scan range of the precursor ion was from 300 to 1800 m/z. The resolution was set to 70,000 for MS scans and 17,500 for the data-dependent MS/MS scans.

### Bioinformatics analysis

For protein identification, we used the Mascot 2.2 program (MatrixScience, RRID: SCR_000307, URL: http://www.matrixscience.com/) to search fragmentation spectra against a human database. For full MS or MS/MS spectra searches, an error of six parts per million (ppm) or 20 ppm was set, respectively, and two missed cleavages were allowed. The peptide mass tolerance was set to 0.5 Da and the fragment mass tolerance was set to 10 ppm. Protein identification was considered valid if at least one peptide was identified with a significance of *P* < 0.05; the proteins that did not satisfy these criteria were rejected. The threshold for accepting MS/MS spectra was a false discovery rate (FDR) of 0.05. A network model of protein interactions according to known protein-protein interactions was made using Retrieval of Interacting Genes (STRING, RRID:SCR_005223, URL: http://string.embl.de/).

### Co-immunoprecipitation (co-IP)

Affinity-purified antibody was bound to AminoLink Plus Coupling Resin (Pierce, Rockford, IL, USA) in a spin column. After preparing the resin column, ice-cold IP lysis/wash buffer was added to the cells. The bait (Annexin A2), prey (AcGal-1), and controls were prepared and added to the resin along with the cell lysate. The mixture was incubated with gentle rocking at 4 °C for 18 h or overnight. The spin columns were centrifuged, and the sample was washed two more times with 200 µl IP lysis/wash buffer, with a centrifugation step after each wash. A 50 µl volume of elution buffer was added to the column in the tube, followed by incubation for 5 min at room temperature. The tube was centrifuged and the flow-through was collected and analyzed by western blotting.

### Immunofluorescence microscopy

Macrophages were seeded on coverslips in a 6-well plate and treated with His-AcGal-1. Lactose (100 mM) served as a negative control. A total of 10^6^ cells were fixed with 4% paraformaldehyde for 15 min at 37 °C. To reduce non-specific interactions, the cells were blocked with 10% BSA for 3 h. The cells were then incubated for 3 h at 37 °C with rabbit anti-human Annexin A2 antibody (Cat# 8235, RRID:AB_11129437; Cell Signaling Technology, Boston, USA) (1:100), followed by fluorescein (FITC)-conjugated anti-rabbit antibody (Cat# 711-095-152, RRID:AB_2315776); Jackson ImmunoResearch Labs, Lancaster, CA, USA (1:200)) and Rhodamine (TRITC)-conjugated mouse anti-His (to detect His-tagged Gal-1) antibody (Cat# 715-025-151, RRID:AB_2340767; Jackson ImmunoResearch Labs, Lancaster, CA, USA) (1:200). The cells were washed, and a drop of cell suspension was placed on a glass microscope slide along with ProLong anti-fade mounting medium (Thermo Fisher Scientific, Waltham, MA, USA). Images of FITC and TRITC fluorescence were acquired on a microscope. To confirm antibody specificity, primary antibodies were omitted in control samples.

### RNA interference

All of the small interfering RNAs (siRNA) used in this study were designed and synthesized by Shanghai GenePharma (Shanghai, China). THP-1 cells, grown to 80% confluence in 6-well plates in 2 ml medium, were treated with PMA 48 h prior to liposome-mediated transfection. Macrophages were transfected with 30 µl siRNA (the final concentration was 300 nM) specific for human *ANXA2* gene (Annexin A2-siRNA, sense 5’-GCA AGU CCC UGU ACU AUU ATT-3’). The negative control siRNA was a scrambled siRNA for *ANXA2* (siNC, sense 5’-UUC UCC GAA CGU GUC ACG UTT-3’). All siRNAs were transfected into cells using GP-Transfect-Mate/Lipofectamine transfection reagent (Invitrogen, Carlsbad, CA, USA). *ANXA2* expression in the transfected cells was tested by quantitative real-time RT-PCR assays, as well as western blotting.

### qRT-PCR

Total RNA from macrophages was extracted using Trizol reagent (Invitrogen). We next used an Agilent Mx3000P qPCR System (Agilent, Santa Clara, CA, USA) in conjunction with One Step SYBR® PrimeScript™ RT-PCR Kit II (Takara Bio, Beijing, China) to analyze viral gene expression. The housekeeping gene *β-actin* was chosen as an internal control, and the data was analyzed by the real-time quantitative PCR software. The sequences of primers used for *ANXA2* and *β-actin* are listed in Table 1. The amplification conditions of real-time quantitative PCR were as follows: 95 °C denaturation for 3 min, followed by 40 cycles of 95 °C annealing for 12 s, 62 °C extension for 40 s.

**Table 1:** Sequences of primers used for real-time PCR

Gene	Primer	Sequence (5′–3′)	Product size (bp)
*β-actin*	Sense	AAACGTGCTGCTGACCGAG	119
	Antisense	TAGCACAGCCTGGATAGCAAC	
*ANXA2*	Sense	ATGGTCTCCCGCAGTGAAGTG	122
	Antisense	AGCAGCGCTTTCTGGTAGTCG	

### Human phospho-mitogen-activated protein kinase (pMAPK) array

The Human Phospho-MAPK Array Kit (Cat# ARY002B; R&D System, Minneapolis, MN, USA) was used according to the manufacturer’s instructions to identify proteins phosphorylated upon AcGal-1 treatment. Briefly, 2 ml of Array Buffer 5 was pipetted into each well of a 4-well plate, which was then incubated for 1 h on a rocking platform shaker. Samples were prepared by adding up to 400 µl of sample to separate tubes. The final volume was adjusted to 1.5 ml with Array Buffer 1. A 20 µl volume of reconstituted detection antibody cocktail was added to each sample, followed by mixing and incubation at room temperature for 1 h. Array Buffer 5 was removed by aspiration and the prepared sample/antibody mixture was incubated overnight at 2 °C on a rocking platform shaker. After washing the membrane with 1× wash buffer for 10 min on the shaker, 2 ml of diluted streptavidin-horseradish peroxidase was pipetted into each well followed by incubation for 30 min at room temperature. Finally, 1 ml of Chemi Reagent Mix was pipetted onto the membrane, which was exposed to X-ray film for 1–10 min.

### Western blot analysis

Cells were treated with 50 μg/ml recombinant AcGal-1 for 30 min and activation of intracellular signaling pathways was evaluated by western blotting; samples treated with 100 µg/ml BSA or left untreated served as negative controls. Cells were lysed in radioimmunoprecipitation assay (RIPA) buffer and centrifuged, 10,000×*g* for 5 min, and proteins in the supernatant were quantified with the bicinchoninic acid assay (BCA) (Beyotime Institute of Biotechnology, Shanghai, China).

Total protein was lysed by RIPA and quantitated by BCA assay. Proteins were separated on a 10% Bis-Tris PAGE gel in Tris-HCl buffer and transferred to a PVDF membrane that was incubated with antibodies against B cell lymphoma (Bcl-2) (Cat. # 4223, RRID:AB_1903909; Cell Signaling Technology, Boston, MA, USA), Bcl-2-associated X protein (Bax) (Cat# 5023, RRID:AB_10557411; Cell Signaling Technology, Boston, MA, USA), cleaved-caspase-3 (Cat# 9661, RRID:AB_2341188; Cell Signaling Technology, Boston, MA, USA), cleaved-caspase-9 (Cat# 9501, RRID:AB_331424; Cell Signaling Technology, Boston, MA, USA), and glyceraldehyde 3-phosphate dehydrogenase (GAPDH; loading control) (Cat# AT0002; CMC-TAG, San Diego, CA, USA) for 2 h at room temperature. The membrane was developed by enhanced chemiluminescence (ECL) using ECL Plus reagent (Bio-Rad, Hercules, CA, USA).

Cell were treated with SP600125 (Cat#S1460; Selleck Chemicals, Houston, TX, USA) to block JNK phosphorylation and MAPK signaling was analyzed by western blotting. Extracellular signal-regulated kinase (ERK)1/2 (pERK1/2) (Cat# 4370, RRID:AB_2315112; Cell Signaling Technology, Boston, MA, USA) phospho-c-Jun N-terminal kinase (pJNK) (Cat# 4668, RRID:AB_823588; Cell Signaling Technology, Boston, MA, USA), Bcl-2, Bax, and cleaved-caspase-3 and -9 as well as GAPDH were detected by western blotting as described above. The grayscale value of signal intensity for each protein was determined using ImageJ software (National Institutes of Health, Bethesda, MD, USA), and the ratio of target protein to GAPDH was calculated.

### Statistical analysis

Data are presented as the mean ± standard deviation. Statistical analyses were performed using Prism v.5.0 (GraphPad, La Jolla, CA, USA) and SPSS v.20.0 (SPSS Inc., Chicago, IL, USA) software. Differences between groups were evaluated by a one-way analysis of variance (ANOVA) followed by the LSD, and *P* < 0.05 was considered statistically significant (Additional file 1: Table S1).

## Results

### AcGal-1 inhibits macrophage viability

**Fig. 1:**
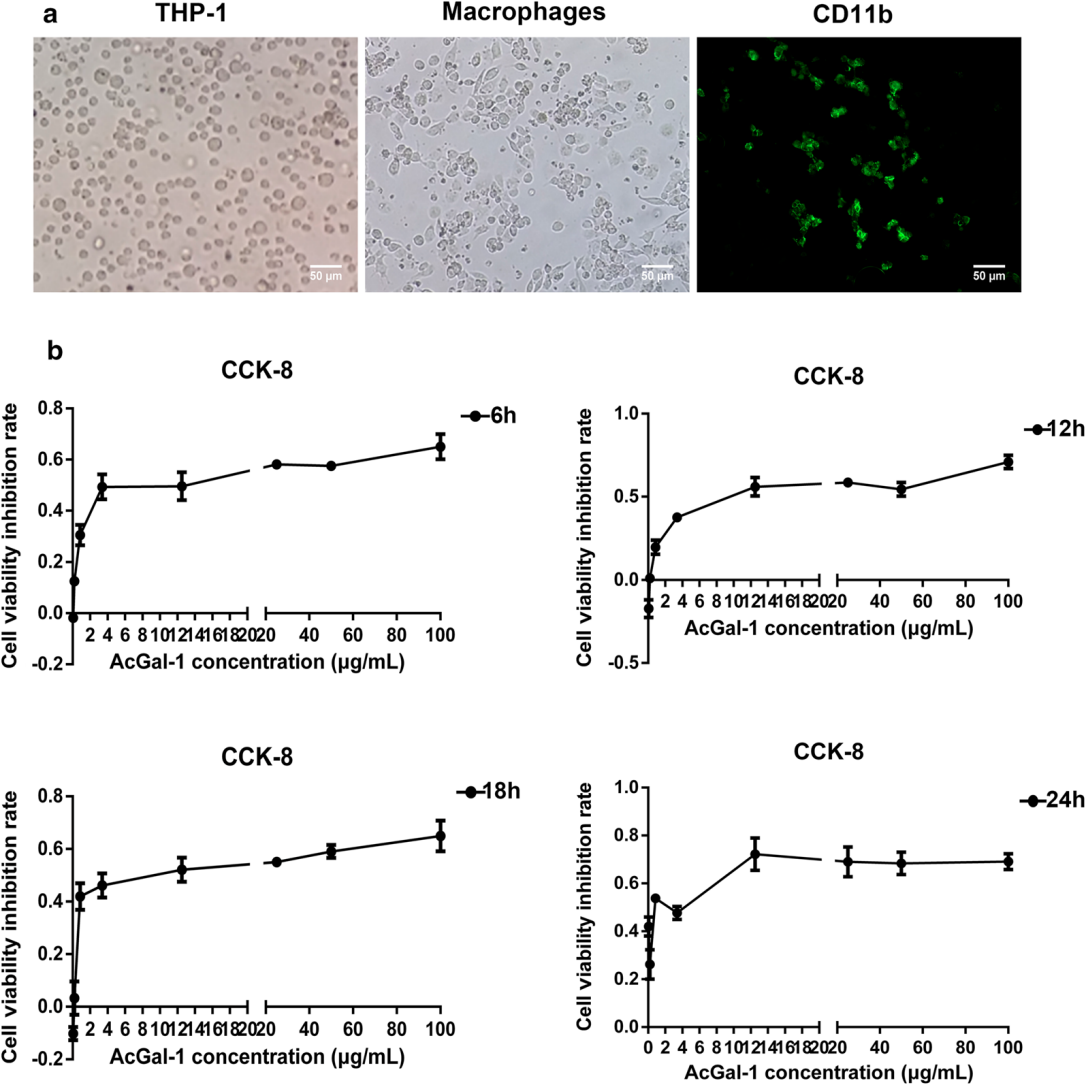
AcGal-1 decreases the viability of THP-1-derived macrophages. **a** PMA treatment of THP-1 cells (left) induced the differentiation into macrophage-like cells (middle). Immunofluorescence staining of CD11b (as a marker for monocyte-derived macrophages) expression on macrophage-like cells (right). **b** The effects of AcGal-1 concentration and treatment time on macrophage viability were evaluated with the CCK-8 assay. Data represent the mean ± standard deviation (SD; vertical bars) of triplicate experiments. *Scale-bars*: 50 µm

We expressed the His-AcGal-1 fusion protein in *E. coli*, then purified and quantitated this protein [23]. To investigate the effects of AcGal-1 on macrophage viability, we used the THP-1 monocyte cell line and induced its differentiation *in vitro*. A microscopic examination of THP-1 cells in suspension revealed that these cells were round, translucent, uniform in size, and occasionally formed clusters. PMA induction altered cell morphology, with an increase in volume and protrusion of pseudopodia, and these cells were able to adhere to the walls of the culture dishes (Fig. 1a). The myeloid differentiation marker, CD11b, was highly expressed in THP-1 upon treatment with PMA [24, 25], suggesting that PMA induced the differentiation of THP-1 cells into macrophage-like cells.

Using the CCK-8 assay, we observed that AcGal-1 decreased the viability of differentiated THP-1 cells. Treatment with 100 μg/ml AcGal-1 for 6 h decreased survival to approximately 70% of that in the control group (Fig. 1b). About 50% of the cells survived (IC_50_ = 49.8) after treatment with 49.8 µg/ml AcGal-1. We therefore used an AcGal-1 concentration of 50 µg/ml in subsequent experiments. The results of the CCK-8 assay also showed that the cell viability was stable at 12 and 18 h, and the latter was therefore selected as the treatment duration.

### AcGal-1 induces macrophage apoptosis/necrosis

**Fig. 2:**
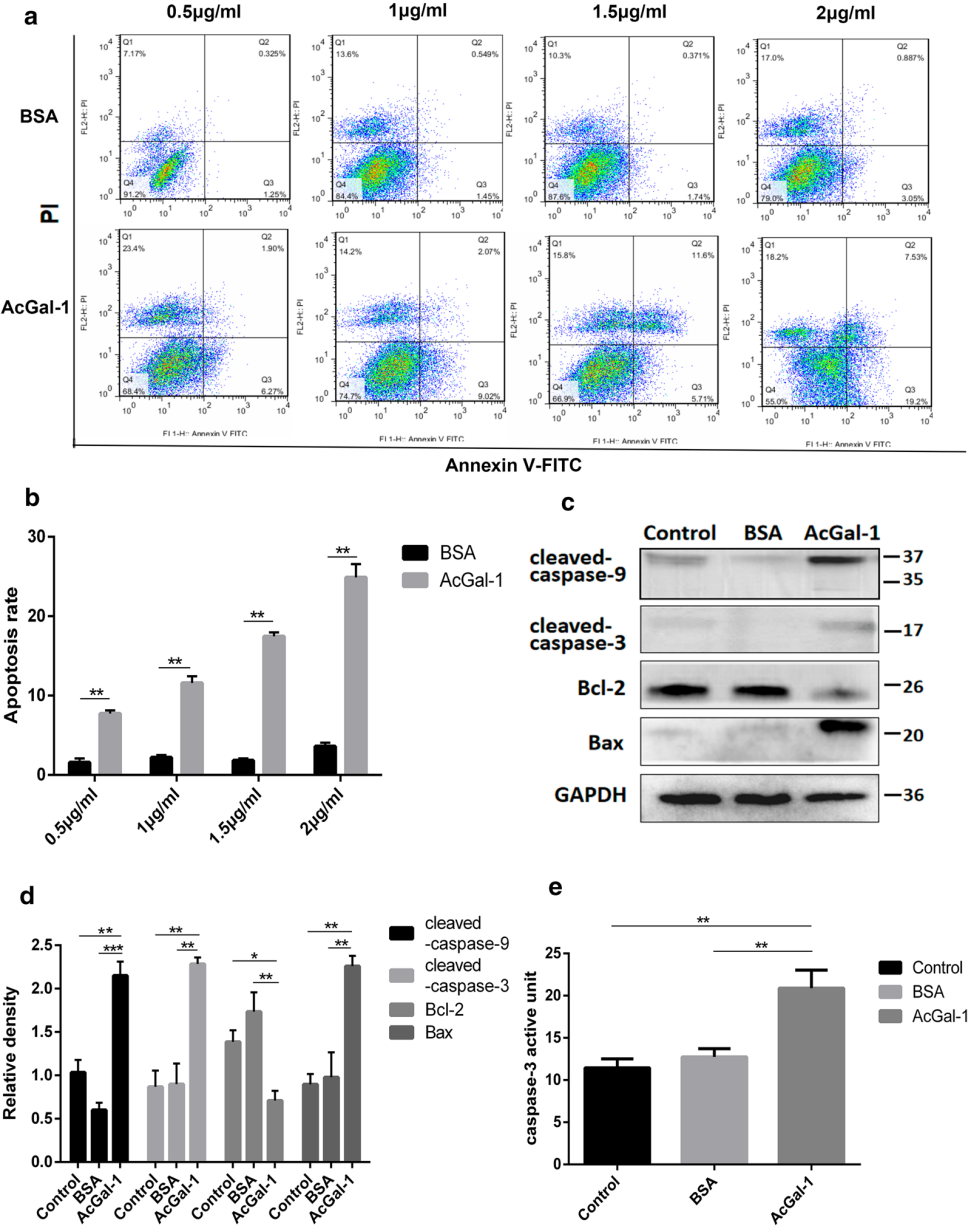
AcGal-1 induces macrophage apoptosis. **a** Flow cytometry analysis showing that apoptosis (revealed by the levels of Annexin V and PI) in AcGal-1-treated macrophages was higher than that in BSA-treated cells (control). **b** Quantitation of the percentage of Annexin V^+^ cells. **c** Expression levels of proteins associated with apoptosis/anti-apoptosis as determined by western blot in different cell groups (control, BSA and AcGal-1). AcGal-1 induced macrophage apoptosis by increasing the levels of cleaved caspase-3, -9, Bax, and decreasing the expression level of Bcl-2. **d** Quantitation of expression intensity of each protein. **e** AcGal-1 increased the enzymatic activity of caspase-3 in macrophages, treated as in **b**. Data represent the mean ± standard deviation (SD; vertical bars) of triplicate experiments. *P < 0.05, **P < 0.01, ***P < 0.001

The reduced viability of macrophage upon treatment with AcGal-1 led us to ask if AcGal-1 might induce macrophage apoptosis. Flow cytometry analysis revealed that the percentages of both early stage apoptotic cells (Annexin V+/PI-) and late apoptotic cells (Annexin V+/PI+) in the AcGal-1-treated group were higher than in cells treated with BSA (Fig. 2a, b). The other cells were living cells (Annexin V-/PI-) or necrotic cells (Annexin V-/PI+) and the latter was also slightly increased to some degree in cells treated with AcGal-1. Western blot analysis revealed that the expression of several apoptosis- or anti-apoptosis-related markers was altered by AcGal-1 treatment (Fig. 2c, d). Compared to levels in the control and BSA groups, the relative expression levels of cleaved-caspase-3 and -9 and Bax were upregulated (2.28 ± 0.13%, 2.15 ± 0.28%, and 2.26 ± 0.21%, respectively), whereas the expression of the anti-apoptotic factor Bcl-2 was downregulated. We further evaluated the enzymatic activity of caspase-3 in macrophages treated with AcGal-1 (or BSA as a control) using Ac-DEVD-pNA. We found that AcGal-1 increased the activity of caspase-3 (28.85 ± 1.72%) relative to untreated cells (11.44% ± 1.06%) and those treated with BSA (12.74 ± 0.98%) (Fig. 2e). Taken together, these results indicated that AcGal-1 induced the apoptosis of macrophages.

### AcGal-1 interacts with Annexin A2 expressed on the macrophage plasma membrane

**Fig. 3:**
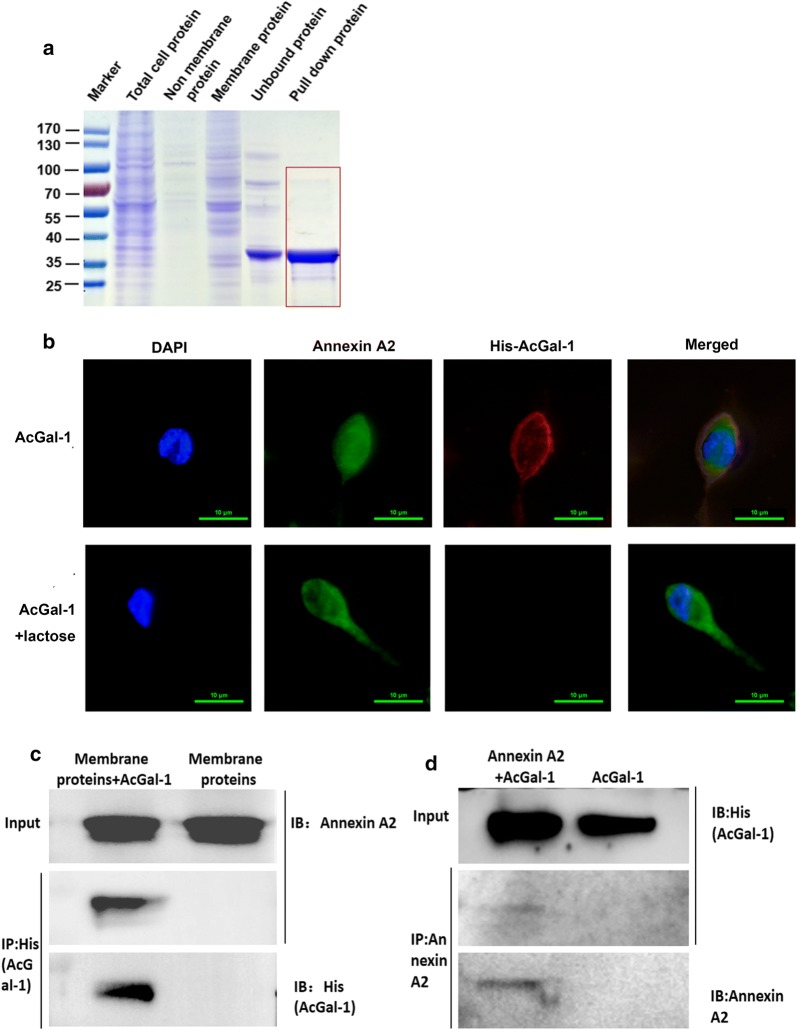
Annexin A2 on the macrophage cell surface interacts with AcGal-1. **a** The different types of proteins were analyzed by sodium dodecyl sulfate-polyacrylamide gel electrophoresis and visualized by Coomassie brilliant blue staining. The “pull-down protein” (red box) that may interact with AcGal-1 was obtained using His-Tag-based immunoprecipitation assay. **b** Immunofluorescence detection of cell surface Annexin A2 and colocalization with AcGal-1, which was abolished by treatment with the glycosylation inhibitor lactose. Macrophages were immunolabeled for Annexin A2 (revealed by FITC, green) and stained for AcGal-1 with (TRITC, red), and then examined using confocal microscopy. **c**, **d** Interaction of Annexin A2 and AcGal-1 were verified by IP, followed by western blot detection of Annexin A2 and AcGal-1. **c** Resin with affinity-purified His-antibody was incubated with AcGal-1-treated or untreated macrophage cell lysates. The Annexin A2 that interacted with His-AcGal-1 was pulled down along with His-mediated immunoprecipitation (IP) and revealed by immunoblot (IB) with the Annexin A2 antibody. AcGal-1 was blotted using the anti-His antibody. **d** Reverse co-IP confirmed the interaction between AcGal-1 and Annexin A2. AcGal-1-treated macrophage cell lysates were incubated with or without the resin bound by affinity-purified Annexin A2-antibody. AcGal-1 that interacted with Annexin A2 was pulled down along with Annexin A2-mediated IP and revealed by IB with the anti-His antibody. Annexin A2 was blotted using the Annexin A2 antibody. *Scale-bars*: **b**, 10 µm

As an exogenous glycoprotein, AcGal-1 is likely to bind to the receptors on the cell membrane. To investigate the molecular basis for the impact of AcGal-1 on macrophages apoptosis, lysates from macrophages from the THP-1 cell line were incubated with His-tag fused to AcGal-1 antibody-conjugated beads and bound proteins were eluted. We carried out co-IP under stringent conditions to pull down only the proteins that strongly interacted with AcGal-1. Several proteins were found in the AcGal-1 precipitate of macrophages (Fig. 3a). The precipitated proteins were then analyzed by LC-MS (Table 2); 26 cell membrane proteins contained more than two unique peptide counts. Among these, three peptides of human Gal-1 were identified as being *A. cantonensis* Gal-1 by BLAST alignment (Table 3). Bioinformatics analysis of these cell membrane proteins with STRING predicted that AcGal-1 may interact with Annexin A2 (Additional file 2: Figure S1). In addition, previous studies have reported that membrane Annexin A2 contains galactoside oligosaccharide residues [26, 27]. Thus, we hypothesized that AcGal-1 with a carbohydrate recognition domain could bind to Annexin A2.

**Table 2:** Mass spectrometry-based identification of membrane proteins obtained by IP

Protein ID	Gene	Uniprot identifier	Species	Protein MW	PI	UniquePepCount	SeqCoverage (%)
Annexin A2	*ANXA2*	sp|P07355|	Human	38603.6	7.57	8	20.94
Adenylyl cyclase-associated protein 1	*CAP1*	sp|Q01518|	Human	51900.86	8.24	7	13.47
Elongation factor 1-alpha 1	*EEF1A1*	sp|P68104|	Human	50140.28	9.1	8	14.94
Annexin A6	*ANXA6*	sp|P08133|	Human	75872.41	5.42	6	9.66
Heat shock protein HSP 90-beta	*HSP90AB1*	sp|P08238|	Human	83263.22	4.97	6	7.04
Hemoglobin subunit alpha	*HBA1*	sp|P69905|	Human	15257.36	8.72	6	26.76
Protein disulfide-isomerase	*P4HB*	sp|P07237|	Human	57115.66	4.76	4	6.10
Coiled-coil domain-containing protein 88B	*CCDC88B*	sp|A6NC98|	Human	164807.13	5.09	3	1.49
ATP-binding cassette sub-family F member 3	*ABCF3*	sp|Q9NUQ8|	Human	79743.72	5.95	3	3.53
ATP-binding cassette sub-family A member 13	*ABCA13*	sp|Q86UQ4|	Human	576152.47	6.01	3	0.42
Voltage-dependent anion-selective channel protein 1	*VDAC1*	sp|P21796|	Human	30772.21	8.62	3	13.43
Annexin A1	*ANXA1*	sp|P04083|	Human	38713.8	6.57	3	1.02
Annexin A5	*ANXA5*	sp|P08758|	Human	35936.34	4.94	3	8.75
Moesin	*MSN*	sp|P26038|	Human	67819.26	6.08	3	4.51
Ribosome-binding protein 1	*RRBP1*	sp|Q9P2E9|	Human	152470.38	8.69	3	0.99
Galectin-1	*LGALS1*	sp|P09382|	Human	26152	8.58	3	7.20
ADP/ATP translocase 1	*SLC25A4*	sp|P12235|	Human	33064.1	9.78	2	6.04
Myosin light polypeptide 6	*MYL6*	sp|P60660|	Human	16929.88	4.56	2	14.57
Neurobeachin	*NBEA*	sp|Q8NFP9|	Human	327817.78	5.78	2	0.48
PH-interacting protein	*PHIP*	sp|Q8WWQ0|	Human	206686.7	9.02	2	0.77
Golgi apparatus protein 1	*GLG1*	sp|Q92896|	Human	134550.64	6.57	2	1.02
Exocyst complex component 1	*EXOC1*	sp|Q9NV70|	Human	101980.39	6.17	2	0.67
E3 ubiquitin-protein ligase UBR4	*UBR4*	sp|Q5T4S7|	Human	573834.61	5.7	2	0.29
Protocadherin beta-4	*PCDHB4*	sp|Q9Y5E5|	Human	87269.06	5.05	2	1.51
Vacuolar protein sorting -associated protein 8 homolog	*VPS8*	sp|Q8N3P4|	Human	161751.77	5.41	2	0.77
Coronin-1A	*CORO1A*	sp|P31146|	Human	51025.7	6.25	2	5.86
ATP synthase subunit alpha, mitochondrial	*ATP5A1*	sp|P25705|	Human	59749.91	9.16	2	3.44

*Notes*: The number of unique peptides detected by UniquePepCount: proteins are arranged from high to low according to the size of UniquePepCount, and the higher the value, the higher the reliability of the protein. SeqCoverage: peptide coverage, that is, the proportion of the number of amino acids detected to the full length of the protein (formula: the number of amino acids detected / the number of all amino acids of the protein). The higher the protein coverage, the higher the kurtosis of the protein may be relatively higher in this sample, but there is no absolute quantification standard

**Table 3:** The peptide data, BLAST of AcGal-1 and Annexin A2

Protein ID	Location at protein	Sequence	Length
sp|P09382| LEG1_HUMAN Galectin-1^a^ (OS, *Homo sapiens*; GN, LGALS1)	196–203	LALHFNPR	8
	219–224	WGNEQR	6
	23–29	IQVLVEPDHFK	11
sp|P07355| ANXA2_HUMAN Annexin A2 (OS, *Homo sapiens*; GN, ANXA2)	29–37	AYTNFDAER	9
	158–168	DIISDTSGDFR	11
	82–88	ELASALK	7
	70–76	LMVALAK	7
	19–29	TPAQYDASELK	11
	128–135	WISIMTER	8
	197–204	DLYDAGVK	8
	136–145	TNQELQEINR	10

^a^These peptides were identified as human galectin-1, but BLAST sequence alignment showed that the peptides were closer to AcGal-1

Using immunofluorescence microscopy, we observed co-localization of His-Ac-Gal-1 and Annexin A2 at the plasma membrane (Fig. 3b). Binding of AcGal-1 to the macrophage surface was revealed by a TRITC-labelled antibody, and binding of Annexin A2 to the membrane was demonstrated using a FITC-labelled antibody. The significant co-localization of red fluorochrome and FITC (merge-yellow) illustrated specific interaction of AcGal-1 to Annexin A2 at the macrophage membrane, which was abolished by treatment with lactose. A further co-immunoprecipitation assay was performed to validate the specific binding of AcGal-1 to Annexin A2 (Fig. 3c, d**)**. Macrophage lysates (membrane proteins) were incubated with AcGal-1 that was connected to the His-AcGal-1-antibody-conjugated nickel beads and bound proteins were pulled down using the anti-His antibody. Annexin A2 was observed in these immunoprecipitates (Fig. 3c). Reverse IP was performed by incubating AcGal-1 with macrophage lysates that were first incubated with the Annexin A2 antibody-conjugated resin and bound proteins were pulled down using the anti-Annexin A2 antibody. His-AcGal-1 was detected in these immunoprecipitates (Fig. 3d). These results confirmed the interaction between AcGal-1 and Annexin A2.

### Reduced Annexin A2 expression in macrophages affects AcGa-1-induced apoptosis

**Fig. 4:**
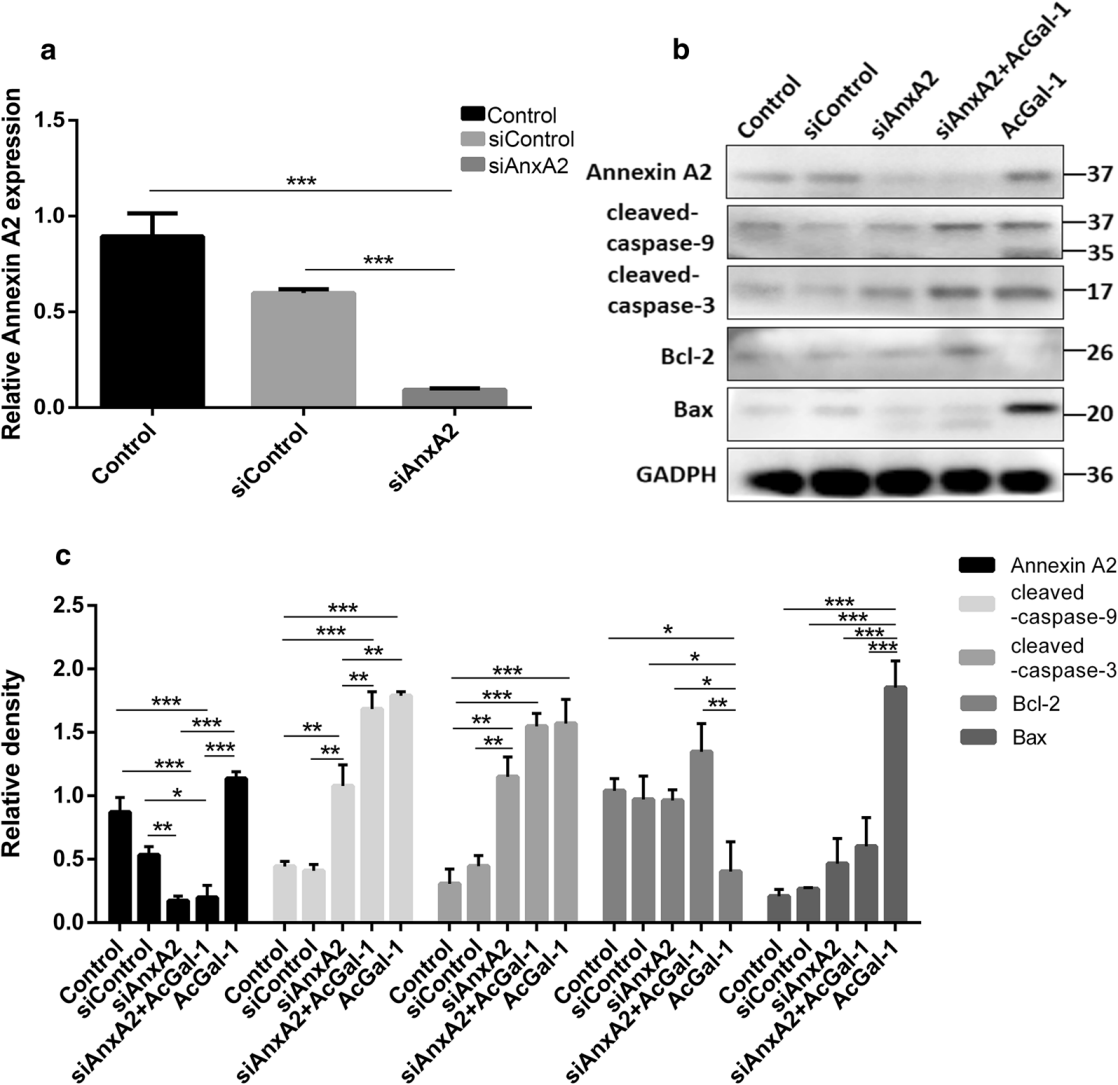
Reduced Annexin A2 expression in macrophages affects AcGa-1-induced apoptosis. **a** Relative expression of *ANXA2* by qRT-PCR in macrophage cells treated with or without indicated siRNAs. **b** The expression of apoptosis-associated proteins Bax, caspase-3, caspase-9 and the expression of anti-apoptotic protein Bcl-2 in indicated groups of cells treated with or without AcGal-1. **c** Quantitation of expression intensity of each protein in **b**. Data represent the mean ± standard deviation (SD; vertical bars) of triplicate experiments. *P < 0.05, **P < 0.01, ***P < 0.001

Next, we investigated whether AcGal-1-induced apoptosis was mediated *via* interaction with Annexin A2 on the macrophage membrane by knocking down Annexin A2 using siRNAs. The qRT-PCR and western blot analysis showed that upon transfection, Annexin A2-siRNA efficiently reduced the level of Annexin A2 compared to control siRNA (siNC) at both RNA and protein levels (Fig. 4a, b). Further western blot analysis showed that AcGal-1 treatment increased the expression of apoptotic proteins caspase-3, -9, and Bax, and reduced the level of Bcl-2, consistent with our above findings (Fig. 4b, c). Compared to control cells, Annexin A2-siRNA-transfected cells did not show significant changes in the expression of caspase-3 and -9, but had significantly reduced Bax and increased Bcl-2 expression after AcGal-1 treatment (Fig. 4b, c), suggesting that reduced Annexin A2 expression in macrophages may prevent macrophages from apoptosis. These results indicated that AcGal-1-induced macrophages apoptosis was at least in part mediated by Annexin A2.

### JNK signaling mediates AcGal-1-induced macrophage apoptosis

**Fig. 5:**
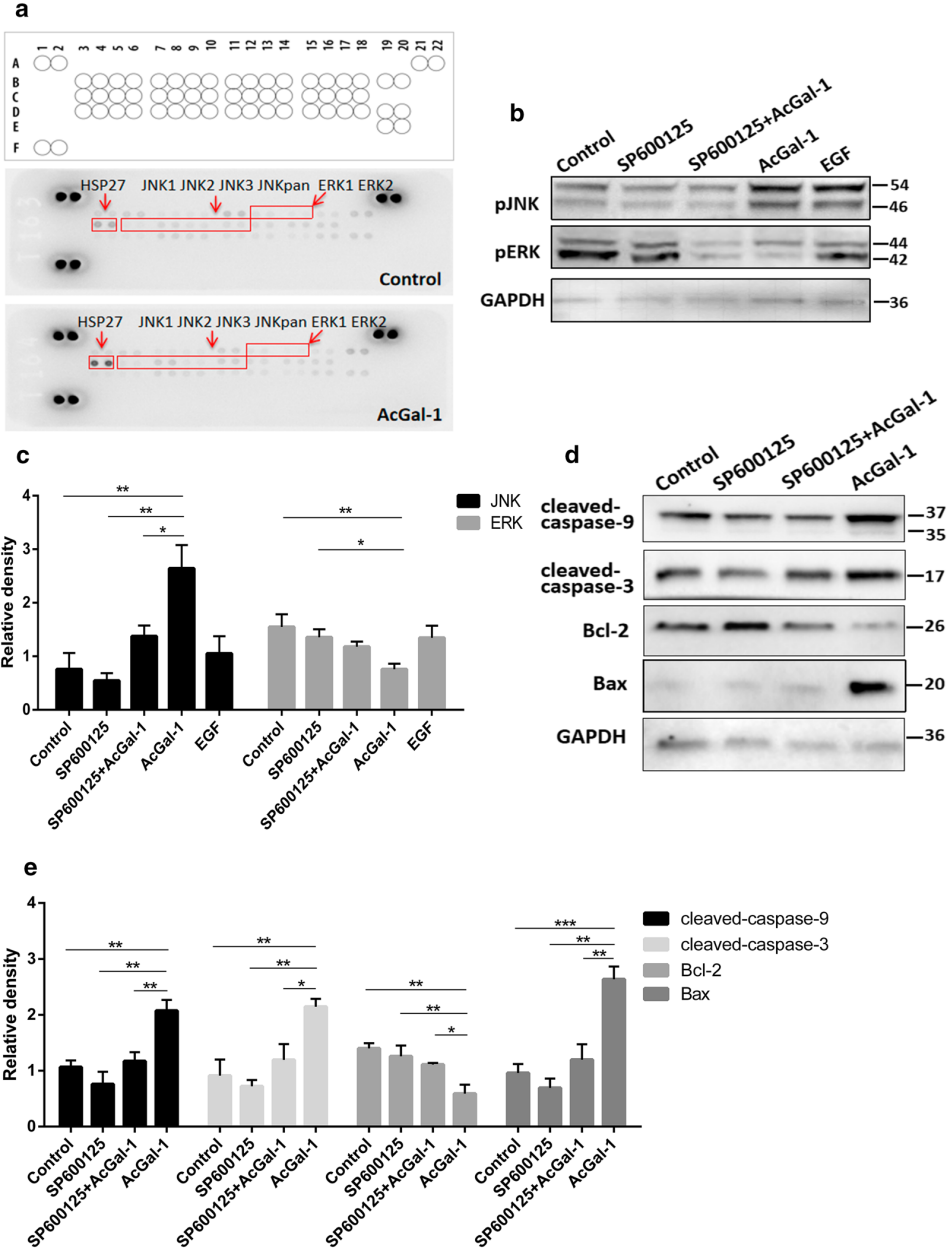
Phosphorylation of MAPK signaling pathway proteins following AcGal-1 treatment. **a** Results of MAPK phospho-antibody microarray. The proteins outlined in a red box showed a significant increase in phosphorylation levels, including JNK and HSP27, and a decrease in pERK1/2, following AcGal-1 treatment. **b** The levels of pJNK and pERK1/2 were confirmed by western blotting. SP600125 was used to inhibit JNK phosphorylation, and cells treated with EGF were used as a positive control to reflect the EGFR/MAPK pathway activation. **c** Quantitation of expression intensity of each protein in **b**. **d** Western blot analysis of apoptosis/anti-apoptosis -related proteins in AcGal-1-treated or non-treated macrophages in the presence or absence of the JNK inhibitor SP600125 on macrophage apoptosis. **e** Quantitation of expression intensity of each protein in **d**. Data represent the mean ± standard deviation (SD; vertical bars) of triplicate experiments. *P < 0.05, **P < 0.01, ***P < 0.001

The Annexin A2-chickpea lectin interaction at the membrane lattice is essential for ligand-induced MAPK pathway-mediated induction of cancer cell apoptosis [28]. To determine whether the interaction between Annexin A2 and AcGal-1 affects MAPK signaling in macrophages, we used a human pMAPK array to screen for phosphorylated proteins in this pathway (Fig. 5a, Table 4). AcGal-1 suppressed the phosphorylation of ERK1/2 while increasing pJNK expression. Western blot analysis using specific phosphorylated antibodies confirmed that compared to control, AcGal-1 reduced the level of pERK1/2 (0.76 ± 0.17 *vs* 1.55 ± 0.41) but increased the expression of pJNK (2.64 ± 0.75 *vs* 0.76 ± 0.53) (Fig. 5b, c).

**Table 4:** Statistical results of MAPK phospho-antibody microarray

Location	Protein description	Control group	Experimental group	(Con-Exp)/Con*% ratio	Regulation type
A1–A2	Reference spots	51937.73	51840.95	0.186338525	None
A21–A22	Reference spots	52784.42	51993.97	1.497506272	None
B3–B4	Akt1	3507.71	3485.56	0.631466113	None
B5–B6	Akt2	3006.91	2396.15	20.31188163	Down
B7–B8	Akt3	1071.16	1027.13	4.110497031	None
B9–B10	Akt pan	997.93	838.29	15.99711403	None
B11–B12	CREB	4019.41	2452.13	18.9927875	Down
B13–B14	ERK1	1344.72	828.83	38.36412041	Down
B15–B16	ERK2	631.38	167.43	73.4818968	Down
B17–B18	GSK-3alpha/beta	1387.87	1173.06	15.47767442	None
B19–B20	GSK-3beta	4955.93	6242.08	− 15.95173862	Up
C3–C4	HSP27	7084.96	16467.05	− 132.4226248	Up
C5–C6	JNK1	1410.1	1672.34	− 18.59726261	None
C7–C8	JNK2	2220.91	3306.2	− 48.86690591	Up
C9–C10	JNK3	1434.32	1113.73	− 22.35135813	None
C11–C12	JNK pan	1679.32	2675.34	− 59.31091156	Up
C13–C14	MKK3	1790.49	1580.41	11.73310099	None
C15–C16	MKK6	1871.84	1560.35	16.64084537	None
C17–C18	MSK2	2274.3	1893.44	16.74625159	None
D3–D4	p38alpha	2133.44	2407.17	− 12.83045223	None
D5–D6	p38beta	1383.76	1283.03	7.279441522	None
D7–D8	p38delta	2690.51	2296.87	14.6306834	None
D9–D10	p38gamma	1932.6	1822.39	5.702680327	None
D11–D12	p53	1426.09	1387.23	2.724933209	None
D13–D14	p70 S6 Kinase	1229.4	1264.16	− 2.827395477	None
D15–D16	RSK1	739.33	722.23	2.312904927	None
D17–D18	RSK2	1989.37	1881.12	5.441421153	None
D19–D20	TOR	2875.41	2469.84	14.10477115	None
E19–E20	NC	0	0		None
F1–F2	Reference spots	52455.55	49893.87	4.883525194	None

*Notes*: By default, the ratio ((Con-Exp)/Con*%)) of greater than ± 20% is considered significant

*Abbreviations*: down, downregulated; up, upregulated

To identify the downstream effectors, macrophages were treated with AcGal-1 and the expression of apoptosis-related proteins was evaluated. Consistently, Bcl-2 was downregulated whereas cleaved-caspase-9 and -3 and Bax were upregulated in the presence of AcGal-1 (Fig. 5d, e). Suppressing JNK phosphorylation with the inhibitor SP600125 decreased the expression of the latter three proteins and restored Bcl-2 to a level similar to that in control cells. These results suggested that JNK phosphorylation might play a critical role in AcGal-1-induced apoptosis of macrophages.

## Discussion

Previous studies have reported the apoptosis and necroptosis of parenchymal and hippocampal astrocytes, neurons, and microglia in mice infected with *A. cantonensis* [2]. However, we do not know the factors playing a role in apoptosis. In our previous proteomic analysis, we discovered expression of galectin-1 protein from *A. cantonensis* (AcGal-1) by two-dimensional electrophoresis (2-DE) combined with immunoblotting assay [29]. AcGal-1 was cloned into a prokaryotic expression vector, expressed, and purified. Western blot analysis showed that the recombinant protein was recognized by sera from mice infected with *A. cantonensis* and the agglutination reaction test indicated that AcGal-1 had good biological activity [23, 30]. The results of the present study provide evidence that AcGal-1 is identified as a new factor for macrophage apoptosis, which may provide explanations for neuro-angiostrongyliasis. Apoptosis is induced *via* activation of caspases including caspase-3, caspase-9, and other apoptosis-associated proteins, while the anti-apoptotic protein Bcl-2 expression inhibits apoptosis [31, 32]. To the best of our knowledge, this is the first report demonstrating that AcGal-1 can induce apoptosis in macrophages derived from THP-1 cells, which are a widely used human monocytic leukemia cell line for monocyte/macrophage differentiation and function [33] owing to various advantages over primary monocytes such as a homogenous genetic background that ensures reproducibility [34].

Nematode galectins have a specific affinity for β-galactosides through an evolutionarily conserved sequence motif [35]. Proteins can be modified into multiple glycoforms with variable binding avidity for Gals; N-glycans are the major Gal ligand at the cell surface [36, 37]. The binding avidity for individual glycoproteins increases with the number of N-glycans per protein [38].

We identified Annexin A2 as a putative binding partner of AcGal-1 by LC-MS and demonstrated that this interaction mediates macrophage apoptosis. Moreover, the interaction of these two proteins was confirmed by immunofluorescence analysis and the impact on macrophage apoptosis by this interaction was evaluated using the siRNA-mediated knockdown of Annexin A2. An earlier study reported that the membrane protein Annexin A2 has a galactosyl side chain [39] with at least one asparagine (N)-linked biantennary mannosyl residue [40]. We propose that the CRD AcGal-1 binds to the N-glycan of Annexin A2, leading to glycosylation and phosphorylation of Annexin A2; the binding of AcGal-1 to Annexin A2 on the cytomembrane of macrophages observed in this study may trigger an intracellular signaling cascade that activates cell apoptosis. Interestingly, we observed that the level of Annexin A2 expression markedly influences AcGal-1-induced apoptosis. In particular, silencing *ANXA2* with siRNA led to a significant decrease in the expression of apoptosis proteins, especially Bax, but increased Bcl-2 expression. This suggests that a decrease in the level of Annexin A2 affected the molecular apoptotic pathway induced by AcGal-1. Although there were no apparent changes in the levels of caspases in this particular experiment, our finding suggests that Annexin A2 is at least partially required for the AcGal-1-induced apoptosis of macrophages.

Annexins are phospholipid-binding proteins conserved in animals and plants that play an important role in membrane/cytoskeleton linkage for exo-/endocytosis [41]; they also participate in the formation of membrane domains *via* their phospholipid-binding function [42]. Annexin A2 functions as a scaffold that organizes signaling proteins to regulate cellular processes such as proliferation, differentiation and apoptosis. Annexin A2 has previously been identified as an EGFR-interacting molecule and the interaction between EGFR and Annexin A2 at the membrane surface is critical for downstream EGFR/MAPK signaling in cells [43]. Annexin A2 is also a substrate of Src; the membrane-bound Annexin A2/EGFR/Src signaling complex activates downstream signaling pathways [44–47]. Additionally, Annexin A2 interacts with Gal-3 at membrane lattices, which is important for EGFR/MAPK signaling in the survival, growth and progression of triple-negative breast cancer [48].

Activated EGFR/MAPK signaling regulates cell proliferation, infiltration, metastasis and angiogenesis and promotes apoptosis [49, 50]. In this study, we found that AcGal-1-induced activation of JNK, a member of the MAPK superfamily that also comprises p38 and ERK [51], could be increased. AcGal-1 activated JNK and induced apoptosis signaling *via* activation of caspase-3 and -9, as determined by western blotting. The pro-apoptotic effect of AcGal-1 in macrophages was diminished by blocking JNK phosphorylation using SP600125, as evidenced by the restoration of cleaved-caspase-9 and -3, Bax, and Bcl-2 levels, supporting that AcGal-1 induces macrophage apoptosis *via* JNK signaling.

In conclusion, we found that AcGal-1 induces macrophage apoptosis *via* interaction with Annexin A2 expressed on the macrophage cell surface and activation of JNK signaling. These findings provide a basis for investigating the pathogenesis and mechanisms of host immune evasion by *A. cantonensis*, which can lead to the development of effective anti-parasite strategies or treatments in cases of infection by this parasite.

## Conclusions

This study investigated the effect of *A. cantonensis* galectin 1 (AcGal-1) on the viability of macrophages and the signaling pathways involved, as well as the interaction between AcGal-1 and Annexin A2 expressed on the cell membrane of macrophages. We found that AcGal-1 interacted with Annexin A2 and induced apoptosis in macrophages, which resulted in reduced viability. AcGal-1 increased the phosphorylation of c-Jun N-terminal kinase (JNK), whereas treatment with the JNK phosphorylation inhibitor SP600125 diminished the apoptosis-inducing effects of AcGal-1. We believe that our study may reveal the mechanisms for *A. cantonensis*-mediated immune evasion and may suggest strategies to prevent or treat cases of *A. cantonensis* infection.

## Supplementary information

**Additional file 1: Table S1.** Statistical comparisons presented in figures.

**Additional file 2: Figure S1.** Bioinformatics analysis of these cell membrane proteins with STRING predicted that AcGal-1 may interact with Annexin A2.

## Data Availability

Data supporting the conclusions of this article are included within the article and its additional files. The datasets used in the present study are available from the corresponding author upon reasonable request.

## Abbreviations

Ac-DEVD-pNA: N-acetyl-Asp-Glu-Val-Asp-p-nitroanilide
AcGal-1: *A. cantonensis* Galectin-1
Bax: B cell lymphoma 2-associated X protein
Bcl-2: B cell lymphoma 2
BSA: bovine serum albumin
co-IP: co-immunoprecipitation
CCK-8: cell counting kit 8
Con: control
CRD: carbohydrate recognition domain
ECL: enhanced chemiluminescence
EGFR: epidermal growth factor receptor
ERK: extracellular signal-regulated kinase
FITC: fluorescein isothiocyanate
Gal-1: galectin-1
IL: interleukin
GAPDH: glyceraldehyde 3-phosphate dehydrogenase
JNK: c-Jun N-terminal kinase
L3: third-stage larvae
LC3-II: microtubule-associated protein 1A/1B-light chain 3-II
LC-MS: liquid chromatography-mass spectrometry
MAPK: mitogen-activated protein kinase
mTOR: mechanistic target of rapamycin
NP40: Nonidet P40
PBMC: peripheral blood mononuclear cell
PI: propidium iodide
PMA: phorbol 12-myristate 13-acetate
RIP: receptor-interacting serine/threonine-protein kinase
THP-1: human acute monocytic leukemia line
TLR: Toll-like receptor
