# Supplementary material for: Angiostrongylus cantonensis Galectin-1 interacts with Annexin A2 to impair the viability of macrophages via activating JNK pathway
Source: Parasit Vectors. 2020 Apr 8;13:183. doi: 10.1186/s13071-020-04038-w (PMC7140382; doi:10.1186/s13071-020-04038-w)
Supplement: Supplementary file 1 — Additional file 1: Table S1. Statistical comparisons presented in figures. [file 13071_2020_4038_MOESM1_ESM.docx]

**Additional file 1: Table S1.** Statistical comparisons presented in the figures

**Figure 1b** **cck-8-6h F(7,16)=69.189, p<0.001**

**cck-8-12h F(7,16)=61.637, p<0.001**

**cck-8-18h F(7,16)=38.423, p<0.001**

**cck-8-24h F(7,16)=12.276, p<0.001**

**Figure 2b** **0.5μg/ml t-test: t(2) =－12.741, P = 0.006**

**1μg/mlt-test: t(2) =－15.923, P = 0.004**

**1.5μg/ml t-test: t(2) =－28.497, P = 0.001**

**2μg/ml t-test: t(2) =－41.696, P = 0.001**

**BSA F(3,8)=315.869, p=0.001**

**AcGal-1 F(3,8)=170.993, p<0.001**

**Figure 2d cleaved-caspase-9** **F(2,6)=36.248,** **p<0.001**

| Comparing groups | P value corrected by LSD |
| --- | --- |
| Control vs BSA | 0.061 |
| Control vs AcGal-1 | 0.001 |
| BSA vs AcGal-1 | 0.000 |

**Figure 2d cleaved-caspase-3 F(2,6)=20.095, p=0.002**

| Comparing groups | P value corrected by LSD |
| --- | --- |
| Control vs BSA | 0.907 |
| Control vs AcGal-1 | 0.001 |
| BSA vs AcGal-1 | 0.002 |

**Figure 2d Bcl-2 F(2,6)=10.322, p=0.011**

| Comparing groups | P value corrected by LSD |
| --- | --- |
| Control vs BSA | 0.179 |
| Control vs AcGal-1 | 0.026 |
| BSA vs AcGal-1 | 0.004 |

**Figure 2d Bax F(2,6)=15.817, p=0.004**

| Comparing groups | P value corrected by LSD |
| --- | --- |
| Control vs BSA | 0.778 |
| Control vs AcGal-1 | 0.002 |
| BSA vs AcGal-1 | 0.003 |

**Figure 2e caspase-3 activity F(2,6)=22.852, p=0.002**

| Comparing groups | P value corrected by LSD |
| --- | --- |
| Control vs BSA | 0.941 |
| Control vs AcGal-1 | 0.001 |
| BSA vs AcGal-1 | 0.001 |

**Figure 4a Annexin A2 siRNA** **F(2,6)=98.444, p<0.001**

| Comparing groups | P value corrected by LSD |
| --- | --- |
| Control vs siControl | 0.002 |
| Control vs siAnxA2 | 0.000 |
| siControlvs siAnxA2 | 0.000 |

**Figure 4c Annexin A2 F(4,10)=27.948, p<0.001**

| Comparing groups | P value corrected by LSD |
| --- | --- |
| Control vs siControl | 0.014 |
| Control vs siAnxA2 | 0.000 |
| Control vs siAnxA2+AcGal-1 | 0.000 |
| Control vs AcGal-1 | 0.040 |
| siControl vs siAnxA2 | 0.009 |
| siControl vs siAnxA2+AcGal-1 | 0.014 |
| siControl vs AcGal-1 | 0.000 |
| siAnxA2 vs siAnxA2+AcGal-1 | 0.819 |
| siAnxA2 vs AcGal-1 | 0.000 |
| siAnxA2+AcGal-1 vs AcGal-1 | 0.000 |

**Figure 4c cleaved-caspase-9 F(4,10)=41.407, p<0.001**

| Comparing groups | P value corrected by LSD |
| --- | --- |
| Control vs siControl | 0.826 |
| Control vs siAnxA2 | 0.001 |
| Control vs siAnxA2+AcGal-1 | 0.000 |
| Control vs AcGal-1 | 0.000 |
| siControl vs siAnxA2 | 0.001 |
| siControl vs siAnxA2+AcGal-1 | 0.000 |
| siControl vs AcGal-1 | 0.000 |
| siAnxA2 vs siAnxA2+AcGal-1 | 0.002 |
| siAnxA2 vs AcGal-1 | 0.001 |
| siAnxA2+AcGal-1 vs AcGal-1 | 0.483 |

**Figure 4c cleaved-caspase-3 F(4,10)=19.414, p<0.001**

| Comparing groups | P value corrected by LSD |
| --- | --- |
| Control vs siControl | 0.488 |
| Control vs siAnxA2 | 0.001 |
| Control vs siAnxA2+AcGal-1 | 0.000 |
| Control vs AcGal-1 | 0.000 |
| siControl vs siAnxA2 | 0.004 |
| siControl vs siAnxA2+AcGal-1 | 0.000 |
| siControl vs AcGal-1 | 0.000 |
| siAnxA2 vs siAnxA2+AcGal-1 | 0.065 |
| siAnxA2 vs AcGal-1 | 0.054 |
| siAnxA2+AcGal-1 vs AcGal-1 | 0.912 |

**Figure 4c Bcl-2 F(4,10)=3.739, p=0.041**

| Comparing groups | P value corrected by LSD |
| --- | --- |
| Control vs siControl | 0.787 |
| Control vs siAnxA2 | 0.763 |
| Control vs siAnxA2+AcGal-1 | 0.248 |
| Control vs AcGal-1 | 0.029 |
| siControl vs siAnxA2 | 0.975 |
| siControl vs siAnxA2+AcGal-1 | 0.163 |
| siControl vs AcGal-1 | 0.046 |
| siAnxA2 vs siAnxA2+AcGal-1 | 0.155 |
| siAnxA2 vs AcGal-1 | 0.049 |
| siAnxA2+AcGal-1 vs AcGal-1 | 0.004 |

**Figure 4c Bax F(4,10)=16.548, p<0.001**

| Comparing groups | P value corrected by LSD |
| --- | --- |
| Control vs siControl | 0.808 |
| Control vs siAnxA2 | 0.301 |
| Control vs siAnxA2+AcGal-1 | 0.124 |
| Control vs AcGal-1 | 0.000 |
| siControl vs siAnxA2 | 0.420 |
| siControl vs siAnxA2+AcGal-1 | 0.183 |
| siControl vs AcGal-1 | 0.000 |
| siAnxA2 vs siAnxA2+AcGal-1 | 0.568 |
| siAnxA2 vs AcGal-1 | 0.000 |
| siAnxA2+AcGal-1 vs AcGal-1 | 0.000 |

**Figure 5c pJNK F(4,10)=7.645, p=0.004**

| Comparing groups | P value corrected by LSD |
| --- | --- |
| Control vs SP600125 | 0.623 |
| Control vs SP600125+AcGal-1 | 0.175 |
| Control vs AcGal-1 | 0.001 |
| Control vs EGF | 0.503 |
| SP600125 vs SP600125+AcGal-1 | 0.078 |
| SP600125 vs AcGal-1 | 0.001 |
| SP600125 vs EGF | 0.257 |
| SP600125+AcGal-1 vs AcGal-1 | 0.013 |
| SP600125+AcGal-1 vs EGF | 0.462 |
| AcGal-1 vs EGF | 0.004 |

**Figure 5c pERK F(4,10)=3.554, p=0.047**

| Comparing groups | P value corrected by LSD |
| --- | --- |
| Control vs SP600125 | 0.448 |
| Control vs SP600125+AcGal-1 | 0.160 |
| Control vs AcGal-1 | 0.006 |
| Control vs EGF | 0.424 |
| SP600125 vs SP600125+AcGal-1 | 0.484 |
| SP600125 vs AcGal-1 | 0.021 |
| SP600125 vs EGF | 0.965 |
| SP600125+AcGal-1 vs AcGal-1 | 0.074 |
| SP600125+AcGal-1 vs EGF | 0.511 |
| AcGal-1 vs EGF | 0.023 |

**Figure 5e cleaved-caspase-9 F(3,8)=10.005, p=0.004**

| Comparing groups | P value corrected by LSD |
| --- | --- |
| Control vs SP600125 | 0.261 |
| Control vs SP600125+AcGal-1 | 0.690 |
| Control vs AcGal-1 | 0.004 |
| SP600125 vs SP600125+AcGal-1 | 0.143 |
| SP600125 vs AcGal-1 | 0.001 |
| SP600125+AcGal-1 vs AcGal-1 | 0.007 |

**Figure 5e cleaved-caspase-3 F(3,8)=8.118, p=0.008**

| Comparing groups | P value corrected by LSD |
| --- | --- |
| Control vs SP600125 | 0.560 |
| Control vs SP600125+AcGal-1 | 0.396 |
| Control vs AcGal-1 | 0.004 |
| SP600125 vs SP600125+AcGal-1 | 0.171 |
| SP600125 vs AcGal-1 | 0.002 |
| SP600125+AcGal-1 vs AcGal-1 | 0.016 |

**Figure 5e Bcl-2 F(3,8)=6.931, p=0.013**

| Comparing groups | P value corrected by LSD |
| --- | --- |
| Control vs SP600125 | 0.484 |
| Control vs SP600125+AcGal-1 | 0.166 |
| Control vs AcGal-1 | 0.003 |
| SP600125 vs SP600125+AcGal-1 | 0.453 |
| SP600125 vs AcGal-1 | 0.008 |
| SP600125+AcGal-1 vs AcGal-1 | 0.025 |

**Figure 5e Bax F(3,8)=16.587, p=0.001**

| Comparing groups | P value corrected by LSD |
| --- | --- |
| Control vs SP600125 | 0.401 |
| Control vs SP600125+AcGal-1 | 0.451 |
| Control vs AcGal-1 | 0.001 |
| SP600125 vs SP600125+AcGal-1 | 0.132 |
| SP600125 vs AcGal-1 | 0.000 |
| SP600125+AcGal-1 vs AcGal-1 | 0.001 |
