# Supplementary figures and images for: Angiostrongylus cantonensis Galectin-1 interacts with Annexin A2 to impair the viability of macrophages via activating JNK pathway
Source: Parasit Vectors. 2020 Apr 8;13:183. doi: 10.1186/s13071-020-04038-w (PMC7140382; doi:10.1186/s13071-020-04038-w)

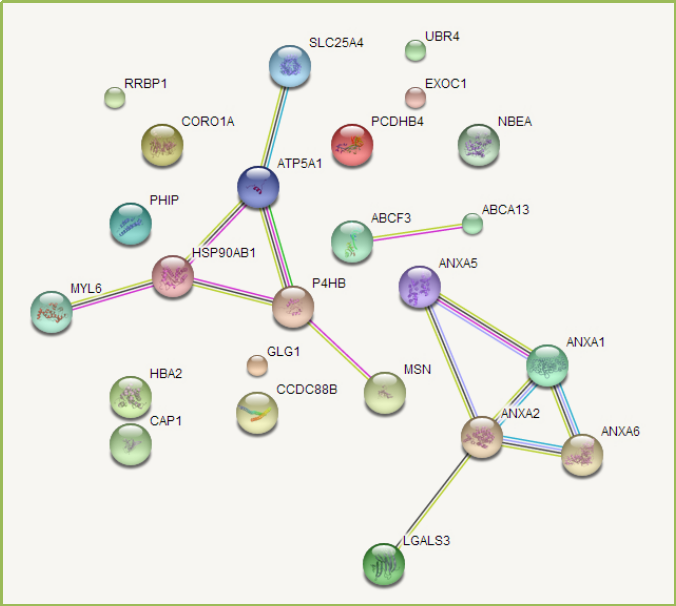

Supplement: Supplementary file 2 — Additional file 2: Figure S1. Bioinformatics analysis of these cell membrane proteins with STRING predicted that AcGal-1 may interact with Annexin A2. [file 13071_2020_4038_MOESM2_ESM.tif]
